# Extent and Implications of the Academia-Industry Connection

**DOI:** 10.4103/0973-1229.32142

**Published:** 2007

**Authors:** Jerome P. Kassirer

**Affiliations:** Tufts University School of Medicine, Editor-in-Chief Emeritus, New England Journal of Medicine, UK

## Introduction

Financial ties between the pharmaceutical, biotechnology and device industries, and the medical profession create conflicts of interest that can damage the integrity of medical information, threaten the well-being of people and inappropriately raise the cost of care. Physicians, like other humans, are susceptible to influence by money. Despite their protestations that they cannot be influenced by lunches, gifts, free trips and even several thousand dollar honoraria, common sense (as well as experiments in cognitive psychology) provide convincing evidence that such arrangements produce a demand for reciprocity (Dana *et al.*, 2003). A physician cannot help being friendly toward a company whose speaker's fees are helping to pay his daughter's college tuition bills.

Reciprocity is often not conscious: more often than not, the need to “pay back” for favours is not recognized by the gift receiver; and given that these urges to reciprocate are subconscious makes them even more insidious. Moreover, though physicians may reject the notion that they can be influenced by money, the mere fact that the pharmaceutical industry spends billions of dollars each year on advertising and that nearly 90 percent of their advertising budget is directed at doctors (through journal ads, drug salesmen, industry-paid speakers) argues that the industry knows exactly that physicians can be influenced (Kerber, 2004).

## The Problem

The extent of involvement of physicians with industry is a closely guarded secret. Physicians are reluctant to release the information and so is industry. Based on required disclosures of financial ties in medical meetings, journal articles and government panels, however, it is apparent that such involvement is quite extensive. Surveys alone suggest that approximately one-quarter of researchers in the life sciences have financial ties with industry (Campbell *et al.*, 1998). At some medical meetings, such as the American Psychiatric Association and ethics guideline panels of the American Heart Association, somewhere between one-third and two thirds of participants acknowledge such arrangements (Anonymous, 2006). Some disclosures list 15 to 20 companies from which single individuals have received honoraria or consulting fees and some medical schools have accepted endowed chairs in the company's name (the Cephalon Professor at Harvard, for example) (Czeisler *et al.*, 2005).

Much less information is available about the extent of financial connections among physicians outside of academic medical centres. Companies recruit “key opinion leaders” in the community to promote their products: sometimes they engage these individuals at educational conferences that consider a certain disease or condition and other times they engage them in clinical research projects centered on one specific therapy. Many believe that such arrangements are merely marketing mechanisms designed to promote the newest and most expensive product. All of the available disclosures provide only a glimpse of the extent of involvement, but, in an unguarded moment, a law firm closely tied to the pharmaceutical industry “spilled the beans”. They said, “It is widely acknowledged that most of the top medical authorities in this country and virtually all of the top speakers on medical topics, are employed in some capacity by one or more of the country's pharmaceutical companies. That is how it should be…” (Popeo *et al.*, 2003). However when one looks at the data, physician involvement with industry in pursuits other than clinical or basic research appears to be extensive.

But involvement of physicians is only the tip of the proverbial iceberg. These industries have developed financial connections (mostly gifts and meals) between nurses, physicians′ office staffs, medical students, house officers and social workers. Major professional organizations solicit and receive thousands (some, hundreds of thousands) of dollars from companies and some allow the companies to dictate how these funds are to be spent. Drug companies also support disease registries, “front organizations” such as the National Anaemia Action Council and the National Initiative in Sepsis Education (NISE) and patient advocacy organizations such as the CHADD (Children and Adults with Attention Deficit Disorder) (Kassirer, 2005). In short, if one adds direct-to- consumer advertising to this list, it is apparent that the industry has permeated a substantial segment of the population and its medicine-related individuals and organizations.

It is legitimate to ask why the permeation of financial ties between pharmaceutical companies and medicine is something to worry about. After all, these industries produce valuable drugs and support medical education, so why raise concerns about the gifts, meals and money that change hands? The first concern is that such arrangements have the potential to introduce bias and thereby to sully the integrity of our database, the very information we rely on to treat patients from day to day. There are many examples in which the integrity of information was questioned because of financial ties (Kassirer, 2004; Gitlin *et al.*, 2004; Stelfox, 1998).

The Endocrine Society's Clinical Practice Guideline for treatment of testosterone deficiency in elderly men was supported by companies that sold testing kits for plasma testosterone and others that sold testosterone replacement preparations. Many of the participants in the guideline development also had personal financial ties with these companies. The panel recommended liberal testing and liberal use of treatment even when the evidence was soft. By contrast, a non-conflicted National Institutes of Health panel reviewed the same information and recommended against widespread testing or treating (Kassirer, 2005). Another panel (of the American Heart Association and American College of Cardiology) that recommended diet, exercise and statins for individuals at high risk for cardiac disease consisted of nine individuals, seven of whom had financial ties (honoraria and speaker's fees) with three to five of the companies that made the statin drugs (Kassirer, 2004). A nearly simultaneous non-conflicted panel from the University of British Columbia found that statins were not beneficial for such patients (Stelfox *et al.*, 1998). In these and countless other examples of synthetic material (guidelines, review articles and editorials), financial conflict of interest seems to have produced biased information which, when applied, could yield benefits to industry but at the same time result in inappropriate prescribing practices.

A second concern about financial incentives is that they raise the cost of care. One salient example is the intravenous use of the drug Natrecor. The U.S. Food and Drug Administration (FDA) approved Natrecor for use only in hospitalised patients with severe cardiac failure. But soon after approval, physicians began to use the drug in their offices for patients with less severe heart conditions despite evidence that kidney failure was an important side effect of the drug. Medicare became alarmed when the charges reached hundreds of millions of dollars in one year (Saul, 2005). Despite a report by a distinguished committee that the drug should be used only as originally approved by the FDA, many physicians continued to prescribe the drug. Though some claimed that they felt “ethically bound” to continue the treatment in outpatients, others wondered whether the $600 fee for the infusion was a driving force (Saul, 2005). This example, though blatant in its implications, pales in comparison to the much greater influence of drug representatives′ gifts, meals and free samples on the cost of care.

Another major concern is that suspicion of doctors′ motives can damage the trust between the public and the profession. Having conflicted physicians on Institutional Review Boards (IRBs) make critical decisions on which studies to approve and having them make decisions on major clinical policies could make the public sceptical that doctors consider their private welfare above that of the public. Most patients still trust their own doctors and this trust is an essential part of the physician-patient relationship. The trust must be preserved.

## The Remedy

What can be done to remedy the problem? Many believe that conflicted physicians should be allowed to participate in decision-making panels such as IRBs, FDA and guideline committees and that their conflicts should be “managed”. By managing conflicts, they propose disclosing the conflicts and recusing those from discussions who have potentially influential conflicts. Some would also prohibit conflicted individuals entirely (Kassirer, 2005). Disclosure alone (allowing conflicted individuals to participate) suffers from an obvious flaw: the receiver of information that a participant is conflicted is in no position to understand whether the conflicted person is being objective or biased (Kassirer, 2006). Recusing individuals from participating in clinical policy decisions is a better strategy. But it requires judgment as to whether the conflict might be sufficiently influential to make a difference and no rules exist to make such determinations. Excluding conflicted individuals completely has its proponents, but many argue that eliminating them eliminates the most knowledgeable experts (Stossel, 2005; Shaywitz, 2006).

I believe that expert policy panels should not include conflicted physicians as decision-makers, but that they should be allowed to testify to the panel about the use of a drug, the performance of a study or the approvability of a drug or device. I believe that there are sufficient numbers of well-trained clinical epidemiologists, data analysts and skilled clinicians similar to those employed in the Cochrane analyses and that these individuals should form the basis for the analysis of clinical policies. It may be difficult to assemble a panel of such nonconflicted individuals, but doing so would eliminate the possible allegation of bias, at least on a financial basis.

I believe that doctors should stop taking gifts and dinners from industry; that they should not be receiving free continuing medical education from industry; that they should not be consulting with industry on marketing issues or developing educational materials for pharmaceutical companies. They should resist joining speaker's bureaus: it is difficult to provide unvarnished information about a company's products when the company is paying one well. A proposal to introduce these principles at academic medical centres (Brennan *et al.*, 2006) has achieved wide acceptance.

Pharmaceutical companies have provided us with valuable drugs and they will continue to do so. I have no objection to physicians in academia engaging in joint research projects with industry or for consulting with industry on scientific issues. But there often exists a fine line between giving scientific advice, which might benefit patients, and marketing advice, which will largely benefit the company. We need much more discussion and guidance about how to make decisions about these difficult grey areas.

## About the Author

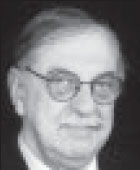



Dr. Jerome P. Kassirer, M.D, Distinguished Professor at Tufts University School of Medicine, has served as governor and regent of the American College of Physicians, chair of the American Board of Internal Medicine and Editor-in-Chief of the New England Journal of Medicine (1991-1999). He is a member of the Institute of Medicine of the National Academy of Sciences, the Association of American Physicians and the American Academy of Arts and Sciences. He has written extensively about health care, for-profit medicine and financial conflict of interest, which are also the subject of his current book, On The Take: How Medicine's Complicity with Big Business Can Endanger Your Health (Oxford).
